# Phenomenology of Gender Dysphoria

**DOI:** 10.1192/j.eurpsy.2025.182

**Published:** 2025-08-26

**Authors:** C. Alves, B. Ferreira

**Affiliations:** Hospital Júlio de Matos, Lisbon, Portugal

## Abstract

**Abstract: Introduction:**

Gender dysphoria (GD) is defined by the presence of clinically significant suffering associated to the marked incongruence between the experienced gender and the one which was assigned at birth. The inherent duplicity to the human condition forces us to reflect on the fact that, in some cases, the body may be the most intimate piece of the Self, whilst also being the most foreign one. The experience of feelings of shame and hatred for oneself and the importance of the Other’s gaze are pressing in GD, which presents as deeply impactful in the individual’s functioning.

**Objectives:**

This review aims to identify and explore the phenomenology of the suffering so often mentioned by GD patients, but which has been ignored and remains mainly unidentified in the psychopathology realm.

**Methods:**

Through the exploration of both the PubMed database and publications by philosophers who have been, throughout the years, approaching the gender theme and distinguishing its evolution along the years, I aim to review the qualitative literature available of the dissection of the different domains of GD.

**Conclusions:**

GD is an ever growing psychiatric diagnostic, frequently presenting with psychiatric comorbidities. Its treatment poses as highly effective, while its consequences may be pervasive and affecting different domains of the individual’s functioning. The exploration of gender identity may be a never ending journey, which makes the acknowledgement of the associated psychopathology fundamental in the design of a truly empathic relationship with these patients.

**Disclosure of Interest:**

None Declared

